# Reproductive performances of the Borgou cow inseminated on natural or induced estrus with semen from Gir and Girolando at the Okpara Breeding Farm

**DOI:** 10.14202/vetworld.2018.693-699

**Published:** 2018-05-25

**Authors:** Foukpê Zhaïrath Adambi Boukari, Ibrahim Traoré Alkoiret, Soumanou Seibou Toléba, Athanase Ahissou, Fataou Zacharie Touré, Aliyassou Mama Yacoubou, Gabriel Assouan Bonou, Ignace Ogoudanan Dotché, Victoire Akpaki, Issaka Youssao Abdou Karim

**Affiliations:** 1 Department of Sciences and Techniques of Animal and Halieutic Production, Faculty of Agronomic, University of Parakou, Parakou, Benin; 2 Department of Animal Production, Faculty of Agronomic Sciences, University of Abomey-Calavi, Abomey-Calavi, Benin; 3 Directorate of Livestock, Milk and Meat Sector Support Project, Okpara Breeding Farm, Benin; 4 Laboratory of Animal Biotechnology and Meat Technology, Department of Animal Production and Health, Polytechnic School of Abomey-Calavi, University of Abomey-Calavi, Abomey-Calavi, Benin

**Keywords:** artificial insemination, Benin, cattle, reproductive performances

## Abstract

**Aim:**

The current study aims to evaluate the reproductive performances of the Borgou cow inseminated on natural or induced estrus with semen from Gir and Girolando at the Okpara Breeding Farm.

**Materials and Methods:**

Semen from exotic breeds was used to inseminate 70 Borgou cows on induced estrus with the norgestomet implant and 285 others on natural estrous. Data on the reproductive performances of inseminated cows were collected.

**Results:**

In inseminated cows on induced estrus, the pregnancy rate was 30% and that of abortion was 9.52%. The fertility rate was 28.57% and those of live births and mortality were, respectively, 105.26% and 5% in these cows. As for inseminated cows on natural estrus, the pregnancy rate was 75.79% and the one of calving was 88.89%. The fertility rate recorded with natural estrous was 66.67% and was significantly higher than the one recorded with insemination on induced estrus. The live births and the birth-weaning mortality rates were, respectively, 98.96% and 11.58% in inseminated cows on natural estrus.

**Conclusion:**

Reproductive performances are better in Borgou cows inseminated on natural estrus than in those inseminated on induced estrus.

## Introduction

Benin livestock counts 2,166,000 cattle, 1,716,000 goats, 860,000 sheep, 414,000 pigs, and 18,198,000 poultry [1]. It provides the national population with 23,431,000 tons of meat and offal, 12,522 tons of eggs, and mostly 107,310 L of milk per year [1]. Despite the size of the national livestock, milk and meat production does not cover the population expressed needs, and this deficit is made up by imports. In 2014, the volume of imported milk was 229,831 tons and that of meat was 281,394 tons [2]. For this external dependence limitation, national production must be increased, and hence, the Benin State’s will to modernize breeding systems. To achieve this, the Livestock Development Project (Phase III), carried out from 2000 to 2006, has introduced the Girolando dairy cows at Kpinnou Breeding Farm to increase the country milk production level [3]. These animals were imported from Brazil and installed at Kpinnou Breeding Farm for local climatic condition adaptation phase, and today, the breed is in full diffusion in peri-urban farming [4]. The Okpara Breeding Farm, for its part, has been specialized on the genetic improvement of the Borgou breed by selection and crossbreeding with exotic breeds Gir, Girolando, and Holstein.

The objectives which motivated the choice of Borgou breed were the improvement of growth and milk production performances. To reach these objectives, it has been set up a selection scheme that gave the expected results [5]. The crossbreeding had the same objectives as the selection. For its implementation, semen from exotic breeds Gir, Girolando, and Holstein was used to inseminate Borgou cows at Okpara Breeding Farm. The artificial insemination (AI) practice requires skilled personnel, fairly sophisticated equipment, and excellent organization. All these requirements influence not only the pregnancy rate but also the other reproductive parameters of the inseminated female. AI is performed on induced estrus using specific hormones or on natural estrus.

The hypothesis of this study was that AI on natural or induced estrus improves the reproductive performances of Borgou females. This study aims to evaluate the reproductive performances of the Borgou cow inseminated on natural or induced estrus at the Okpara Breeding Farm.

## Materials and Methods

### Ethical approval

As the research was artificial insemination study in cattle, Ethical Committee approval was not required.

### Informed consent

The data collection was carried at Okpara Breeding Farm and the people involved are co-authors of the article.

### Study area

The Okpara Breeding Farm is located in the Department of Borgou, township of Tchaourou, district of Kika (2°39-2°53 East longitude and 9°6-9°21 North latitude). It is located on the east of Parakou at 15 km from the township and covers an area of 33,000 hectares, of which about 10,000 are actually exploited. The climatic is of Sudanian type characterized by a succession of a rainy season (May-October) and a dry season (November-April). The average rainfall is 1125 mm per year, and the average annual temperatures vary between 26°C and 27°C. On December 31, 2016, the cattle population of the farm was 709, including 493 Borgou, 85 crossbreed Gir x Borgou, 1 crossbreed Girolando x Borgou, 85 Girolando, 2 crossbreed Holstein x Borgou, 34 Azawakh, and 10 crossbreed Azawak x Borgou. The relief is made of a crystalline peneplain which has hills with very hard rocks. There are large depressions that favor rainwater mobilization toward Okpara’s river and its tributary the Dama. The soil texture is sandy, sandy clay, or limous in some places and supports savanna vegetation dominated by *Andropogon gayanus*.

### Breeding management

Cattle of the Okpara Breeding Farm are reared under a semi-improved system. The monitoring of the cattle is assured by Peulh cowherds led by a team leader. Animals are housed in cowshed constructed of final materials with a tiled roof and concrete floors and in night parks with managers and watering places. They are separated by age class and sex. Feeding is based on natural pastures (*A. gayanus, Leucaena leucocephala*, and *Stylosanthes* spp.), artificial grasslands (*Panicum maximum C1, Brachiaria ruziziensis, and Aeschynomene histrix*), food supplements, and minerals. In the dry season, animals glean their food on natural rangelands, artificial grasslands, and harvest fields where they eat crop residues made of maize straw, cotton residues, groundnut, and cowpea tops. In this season, they also recourse to forest grazings such as *Khaya senegalensis, Afzelia africana*, and *Pterocarpus erinaceus* and forage reserves (hay and ensilage). Animals are conducted to pasture during the day. When returned in the evening, they are enclosed in a cowshed or in a park where they receive water and salt lick *ad libitum*. Lactating cows receive, in addition, food supplements (cottonseed meal, Veto feed, and crop residues). The Veto food is composed of rice bran, cereals residues, cottonseed and palm meals, butylated hydroxytoluene (hydroxytoluene butylate: Food additive), amino acids, limestone, and dicalcium phosphate. This food is formulated by a Beninese public limited company, Veto Services. In the bromatological plan, this complement is composed of protein matter (10%), fats (0.9%), calcium (0.8%), phosphate (0.5%), and starch (8.5%). Calves <4 months old and weak animals are kept in the park and graze around. Animals’ watering is mainly provided by a water tower and two water reservoirs. Reproduction is mainly based on organized coitus. Animals are split into herds. Each herd is composed of reproductive females (25 in average) and one reproductive male. The Livestock Development Project (Phase III) had set a genetic improvement plan based on selection and crossbreeding.

The sanitary prophylaxis in use respects hygienic rules and consists of daily cleaning of the feed and water containers as well as sweeping of the stabling parks and cowshed.The medical prophylaxis plan is characterized by (a) External deworming in a deeping tank monthly in the rainy season and twice the month in the dry season; (b) internal deworming done 3 times the year; however, in a case of diarrhea, the concerned animal is automatically dewormed; (c) trypano-prevention every 2-3 months with Tripadim^®^ (Diminazene Diaceturate) or Tripamidium^®^ (Isometamidium Chloride) produced by the MERIAL laboratory (France); (d) vitamin therapy by administering VETOQUINOL Stress Vitam (Vitamins A, D3, E, B1, B6, B5, and B3, choline chloride, lysine hydrochloride, and glycine); and (e) vaccinations against foot-and-mouth disease (at the beginning of the dry season), pasteurellosis (at the beginning and at the end of the rainy season), and bovine contagious pleuropneumonia (at the end of the rainy season). Vaccines used are, respectively, Aftovax, Pastovax or Pastobov, and Perivax. Specific treatments are made against occasional diseases depending on the detected clinical cases.

### Methodology

AI was performed on 355 Borgou cows reared at the Okpara Breeding Farm. The semen used was from Gir and Girolando breeds and was stored in liquid nitrogen at −196°C to preserve their quality. During the experiment, AI was done on natural or induced estrus.

AI was performed after estrus induction. Borgou cows of 3-8 years old, weighing more than 200 kg, empty and disease free, were selected as reproductive animals. After the sorting, selected cows were prepared for 1 month during which they underwent an endorectal gynecological examination to detect those with a good genital apparatus for estrus synchronization. They were fed sufficiently (after pasture, they received cottonseed and minerals in complementation) and benefited from regular health monitoring. Once chosen, the subcutaneous implant (norgestomet) was placed on the outer surface of each cow’s ear. 10 days later, they received an injection of prostaglandin. The implant placement took 12 days. At the implant withdrawal, cows received an injection of pregnant mare serum gonadotropin (PMSG), and 48-72 h later, they were inseminated after estrus observation. If 21 days after insemination, new estrus occurred in some cows, and they are considered as having not been fecundated and are then inseminated again. AI on induced estrus was performed in 2003 on 70 cows.

For cows inseminated on natural estrus, the ordinary animal health monitoring was strengthened, and cows selected for AI were isolated from progenitor males. When the estrus is detected, they are inseminated once without any specific treatment (no induction) in the morning for those detected in the evening of the previous day and in the evening for those detected in the morning. AI on natural estrus was done on 285 Borgou cows for 5 successive years from 2003 to 2007.

The feeding of inseminated cows was based on natural and artificial pasture as described above in the breeding mode. These cows are taken to the pasture by a cowherd. They left in the mornings at 9 am and returned in the evenings at 5 pm. When arrived from pasture, inseminated cows received each per day on average 2 kg of cottonseed or Veto Service provender in supplementation. The mineral supplementation was made of salt lick distributed constantly and as desired. The health monitoring of cows was identical to that described above in the breeding mode.

### Data collection

Data were collected from 22^nd^ August to 2^2n^d December 2016 and were extracted from annual activity reports from 2002 to 2007 at the Okpara Breeding Farm. The following data were collected on inseminated cows: The number of inseminated cows, the number of pregnancies recorded, the number of pregnancies recorded after the first AI, the number of pregnancies recorded on returning estrus, number of abortions, number of calving, number of births, the number of live births, and the number of dead calves from birth to weaning.

### Performance rate calculation Collected data were used to calculate the following rates


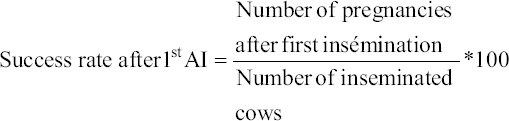



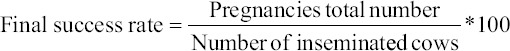



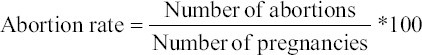



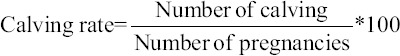



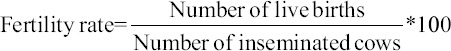



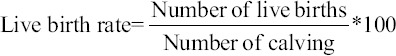



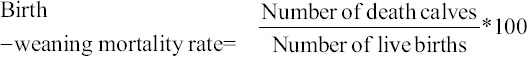


### Statistical analysis

The statistical analysis system [6] software was used for the statistical analysis. For each reproductive performance rate, a confidence interval (CI) of 95% was calculated using the following formula:


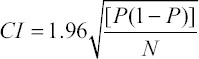


Where *P* is the reproductive performance rate and *N* is the sample size.

From 2003 to 2007, each breeding performance rate was calculated per year and compared pairwise by the bilateral *Z*-test. Reproductive performance was compared between inseminated cows on induced estrus in 2003, on natural estrus in 2003, and on natural estrus from 2003 to 2007. The effect of seasoning, calving, and parity was not considered because of the small sample size, and inseminations were not performed in all the seasons, for all the calving ranks.

## Results

### Reproductive performance of Borgou females artificially inseminated on induced estrus

Table-1 presents the results of AI from induced oestrus in 2003. Out of 70 inseminated cows, 17 became pregnant after the first trial, 4 more after the second insemination (21 in total), and 19 of them calved. Thus, the success rate after the first insemination was 24.28% and the final AI success rate was 30%. A total of 2 inseminated cows aborted resulting in an abortion rate of 9.52%. The fertility rate was 28.57% while the rates of live births and mortality were 105.26% and 5% respectively for the same year.

**Table-1:** Artificial insemination on induced estrus of Borgou cows at the Okpara Breeding Farm in 2003.

Variables	Results
Inseminated cows on induced estrus	70
Inseminated cows after returning estrus	7
Recorded pregnancies	21
Recorded pregnancies after the first insemination	17
Pregnancies after insemination on returning estrus	4
Success rate after the first insemination (%)	24.28±10.04
Final success rate (%)	30±10.73
Abortion rate (%)	9.52±12.55
Calving rate (%)	90.48±12.55
Fertility rate (%)	28.57±10.58
Live birth rate (%)	105.26
Birth-weaning mortality	1
Birth-weaning mortality rate (%)	5±9.55

### Reproductive performance of female Borgou artificially inseminated on natural estrus

The AI on natural estrus results over 2003-2007 is presented in Table-2. In 2003, over 77 inseminated cows, 63 cases of pregnancies were recorded (81.82% of success rate), of which 18 cases recorded after insemination on returning estrus (58.44% of success rate after the first insemination). The final success rate was 82.09% in 2004, but this rate was lower in 2007 (63.16%). No significant differences were found between the success rates after the first insemination during the 5 years. The lowest abortion rate (3.17%) was recorded in 2003 while the highest was registered in 2006 (17.24%) and 2007 (19.44%). The highest calving rate was obtained in 2003 (96.83%) and the lowest in 2007 (80.56%). The highest fertility rates were recorded in 2003 (79.22%) and 2004 (73.13%), while the lowest fertility rates were recorded in 2006 (52.38%) and 2007 (50.88 %). The birth-weaning death rate was lower in 2003 (6.56%) and higher in 2005 (24.14%). The lowest success, fertility, and calving rates were recorded in 2007.

**Table-2:** Artificial insemination on natural estrus of Borgou cows at the Okpara Breeding Farm.

Variables	Results				

	2003	2004	2005	2006	2007
Inseminated cows on natural estrus	77	67	42	42	57
Inseminated cows after returning estrus	26	18	7	5	6
Recorded pregnancies	63	55	33	29	36
Recorded pregnancies after the first insemination	45	45	31	26	32
Recorded pregnancies after insemination on returning estrus	18	10	2	3	4
Success rate after the first insemination (%)	58.44±11.01^a^	67.16±11.24^a^	73.81±13.30^a^	61.90±14.68^a^	56.14±12.88^a^
Final success rate (%)	81.82±8.61^a^	82.09±9.18^a^	78.57±12.41^ab^	69.05±13.98^ab^	63.16±12.52^b^
Abortion rate (%)	3.17±4.33^b^	10.91±8.24^ab^	12.12±11.14^ab^	17.24±13.75^a^	19.44±12.93^a^
Calving rate (%)	96.83±4.33^a^	89.09±8.24^ab^	87.88±11.14^ab^	82.76±13.75^b^	80.56±12.93^b^
Fertility rate (%)	79.22±9.06^a^	73.13±10.61^a^	69.05±13.98^ab^	52.38±15.10^b^	50.88±12.98^b^
Live birth rate (%)	100^a^	100^a^	100^a^	91.67±11.06^a^	100^a^
Birth-weaning mortality	4	5	7	2	4
Birth-weaning mortality rate (%)	6.56±6.21^b^	10.20±8.48^ab^	24.14±15.57^a^	9.09±12.01^ab^	13.79±12.55^ab^

Frequencies of the same line followed by different letters differ significantly at the threshold of 5%

The final success rate of AI on natural estrus in 2003 (81.82%) was higher (p<0.05) than the one recorded after induced estrus (30%) during the same year (Table-3). The tendency was the same for the fertility rate. However, there is no significant difference between abortion, calving, live birth, and birth-weaning death rates in cows inseminated on natural and induced estrus for the same year.

**Table-3:** Comparison of results of artificial insemination performed on natural and induced estrus with Borgou cows at the Okpara Breeding Farm.

Variables	Induced estrus in 2003	Natural estrus in 2003	Natural estrus from 2003 to 2007
Inseminated cows	70	77	285
Inseminated cows a second time after the failure of the first insemination	7	26	62
Recorded pregnancies	21	63	216
Recorded pregnancies after the first insemination	17	45	179
Recorded pregnancies after insemination on returning estrus	4	18	37
Success rate after the first insemination (%)	24.28±10.04^b^	58.44±11.01^a^	62.81±5.61^a^
Final success rate (%)	30±10.73^b^	81.82±8.61^a^	75.79±4.97^a^
Abortion rate (%)	9.52±12.55^a^	3.17±4.33^a^	11.11±4.19^a^
Calving rate (%)	90.48±12.55^ab^	96.82±4.33^a^	88.89±4.19^b^
Fertility rate (%)	28.57±10.58^c^	79.22±9.06^a^	66.67±5.47^b^
Live birth rate (%)	105.26^a^	100.00^ab^	98.96±1.44^b^
Birth-weaning mortality	1	4	22
Birth-weaning mortality rate (%)	5±9.55^a^	6.56±6.21^a^	11.58±4.55^a^

Frequencies of the same line followed by different letters differ significantly at the threshold of 5%

From 2003 to 2007, over 285 cows inseminated on natural estrus of which 62 after returning estrus (Table-3), 216 pregnancy cases were recorded corresponding to a final success rate of 75.79%. This rate was significantly higher than the one recorded with AI on induced estrus (p<0.05). A total of 192 births were recorded, resulting in a calving rate of 88.89%. This calving rate was significantly higher (p<0.05) than that recorded in 2003 with AI on natural estrus. However, there was no significant difference between this calving rate and that recorded with induced estrus. The fertility rate recorded from 2003 to 2007 on natural estrus was 66.67% and was significantly higher than that recorded with insemination on induced estrus. The live birth rate of AI on induced estrus in 2003 was significantly higher than that of insemination on natural estrus from 2003 to 2007. No significant difference was observed in the mortality rates.

## Discussion

### Reproductive performance of Borgou females artificially inseminated on induced estrus

#### Pregnancy rate

The success rate of the AI on induced estrus was 30% at the Okpara Breeding Farm. This rate was initially one of the first successes of the AI in Benin. Within the context of Benin-Nippon cooperation and under the Milk Program of the Breeding Management, AI trials were performed on inducted estrus in 1999. For this, semen from Montbéliarde was used to inseminate the Borgou, Lagunaire, and crossbreed Borgou x Lagunaire in Southern Benin. Among 23 inseminated cows, 7 were pregnant with then a pregnancy rate of 30.43% [7]. This result obtained under the Milk Program is identical to the one of the current study.

The pregnancy rate obtained at the Okpara Breeding Farms is higher than that of 22% obtained with the Azawak at the Sahelian experimental station of Toukounous [8] with the same implant. This difference in success rate could be related to the breeds used receptivity, the inseminator’s experience, and the breeding environment and the females feeding.

Besides, at the Toukounous station, AI on induced estrus success rates of 25.80% and 28.57% was, respectively, reported in Azawak females using the PRID^®^ vaginal spiral and prostaglandin [8]. These rates, although better than those obtained using the Norgestomet implant in the same station, are lower than the one obtained in our study at Okpara. However, the PRID^®^ vaginal spiral and prostaglandin may also be tested under Okpara Breeding Farm’s conditions to verify whether the success rates could also be better than the one of the norgestomet implants as reported in Toukounous station.

In contrast to the AI success rates at Toukounous, the AI on induced estrus success rate at Okpara farm is lower than those obtained in other breeds depending on the implants and the females feeding mode [9-13].

Finally, in semi-intensive breeding farms of the peri-urban area of Ouagadougou in Burkina Faso, Zongo *et al*. [12] reported a pregnancy rate of 42.7% in the Goudali zebus synchronized with the norgestomet implant and inseminated twice. The inferiority of the pregnancy rate recorded at the Okpara farm compared to those reported by the above authors could be explained by the fact that only one insemination was performed on females at this farm, whereas the other females were successively artificial inseminated twice after estrus induction. As the double insemination gives ovum more chance of being fecundated, it is normal that the pregnancy rates obtained by these authors are higher than the one recorded at the Okpara farm.

#### Calving rate and abortion rate

The calving rate (90.48%) and the abortion rate (9.19%) obtained in this study are satisfactory and are not so different from those recorded at the Okpara Breeding Farm for organized coitus, of which the calving rate is 95.65% in 2011 and 93.90% in 2012 with respective abortion rates of 4.31 and 6.10% [3]. These rates are close to those recorded by Yogbare [14] for the calving rate (93.6%) and the abortion rate (6.41%) in Burkina Faso in local breed cows reared in traditional systems.

Byishimo [11] recorded an abortion rate of 2.27% in Girolando females synchronized with PRID® and reared under an intensive system at the agro-pastoral farm of Pout in Senegal. The difference between the abortion rate obtained in the current study and that reported by Byishimo [11] is explained by the breeding mode. Females of the agro-pastoral farm of Pout are in an intensive breeding farm where they are better treated (food supplement, health monitoring) than those of the Okpara farm, of which food supplements and the health monitoring are influenced by a project financing.

#### Fertility and live birth rates

The fertility rate (28.57%) was low in the present study compared to the rates obtained with the organized coitus in the Borgou breed at the same farm in 2011 (41.11%) and 2012 (40.96 %) during this farm cattle breeding inventory [3]. AI on induced estrus must be better managed to achieve a fertility rate close to that of organized coitus.

The live birth rate (105.26%) obtained in our study reveals the presence of a twin birth due certainly to the used hormone effect. This live birth rate is close to the prolificacy rate (106%) recorded by Kouamo *et al*. [13] in a traditional environment with the Gobra zebus after insemination on induced estrus with the PRIDOESTROL vaginal device.

#### Birth-weaning mortality rate

The birth-weaning mortality rate (9.55%) obtained in the current study is higher than those obtained in organized coitus in 2011 (6.31%) and 2012 (6.49%). This difference is related to the crossbreed calves fragility due to a loss of rusticity consecutive to the exotic blood presence in their genetic patrimony. However, this birth-weaning mortality rate of calves produced after estrus induction at the Okpara Farm is close to the rate of 10% obtained by Niang [15] in Eastern Senegal and Upper Casamance and 11.9% recorded by Yugara [16] in Kadiogo, Burkina Faso. On the contrary, it is higher than the 3.13% recorded in the province of Bazèga [14] in Burkina Faso.

### Reproductive performance of females Borgou artificially inseminated on natural estrus

#### Pregnancy rate

The pregnancy on natural estrus global rate from 2003 to 2007 was in average 75.79% and significantly higher than the rate recorded at the same farm after estrus induction. This difference is explained by the fact that Borgou cows may not have been receptive to the hormonal methods used in estrus induction, or after induction, the estrus has been silent, i.e., cows were not inseminated at the best moment. The obtained rate is higher than the one of 44% recorded by Issa *et al*. [8] at the Toukounous station in the Azawak zebus inseminated on natural estrus. It is also higher than the 38% recorded by Niang [15] in the Kolda region of Senegal and even than the rates reported by Ba [17] in different countries of the sub-region: 56% from 1990 to 1996 in Mali; 49% from 1995 to 1998 and 51.93% from 2003 to 2005 in Senegal; and 62% in the Guinea Republic.

#### Calving rate and abortion rate

The calving rate obtained in the current study is 88.89% in cows inseminated on natural estrus. It is identical to the one observed on induced estrus in the present study, and consequently, the estrus induction did not influence the calving rate. These cows inseminated on natural estrus calving rate are higher than the one of 32.6% obtained by Niang [15] in the region of Kolda in Senegal and 28% reported by Ba [17] from 1995 to 1998 in Senegal. However, it is on the other hand close to that reported by Yougbare [14] in Burkina Faso (93.6%) and by Pousga [18] in Mali (97%).

The abortion rate was 11.11% and is close to that recorded (9.19%) in inseminated cows after estrus induction. Estrus induction, therefore, does not influence the abortion rate. The value obtained in cows on natural estrus in the current study is higher than the 6.41% reported in Burkina Faso [14].

### Fertility, live birth, and birth-weaning mortality rates

The fertility rate of females inseminated on natural estrus (66.67%) is higher than the one recorded after estrus induction at the same farm. This difference is explained by the low gestation rate of the AI on estrus induction. Concerning the live birth rate (98.96%), it is lower than the rate of 105.26% recorded with AI after estrus induction. This is certainly due to the superovulation effect of the PMSG used during the estrus induction. Kouamo *et al*. [13] report that the use of PMSG is often associated with twinning in the herd because its action is dose dependent; it induces ovulation (400-500 IU) and/or superovulation (2000 IU).

The birth-weaning mortality rate (11.58%) of calves from inseminated cows on natural estrus is close that recorded with AI on induced estrus. The estrus induction does not then influence calves mortality from the birth to the weaning.

### Reproductive performance evolution over time

AI on natural estrus was carried out from 2003 to 2007. The best success rates were recorded in the first year. Instead of growing with experience acquisition of farm workers, this rate has decreased over time so that the lowest success rate was recorded in 2007. It was the same with the fertility and the calving rates. Conversely, the abortion rate was lowest in the first year and has increased year by year up to its higher level in 2007. The mortality rate was also lower in the first year but higher in 2005. This performance evolution is due to the means given to the farm for the AI realization. Indeed, the inseminations were carried out in 2003 under the Breeding Development Project which was at its beginning and therefore gave more financial means to the farm activities. In 2007, the project was at its end and resources had to be reduced.

The highest live birth rate with the AI on natural estrus is 100% and means that no twin births have been observed. This confirms the PMSG effect mentioned with insemination on induced estrus.

## Conclusion

This study has evaluated the reproductive performance of the Borgou cow inseminated on natural or induced estrus. The success and fertility rates recorded on natural estrus are significantly higher than those recorded with insemination on induced estrus. The feeding and breeding conditions also favor the success of the AI. A comparison of the growth and milk production performances of the Borgou breed with that of the crossbreeds Gir x Borgou and Girolando x Borgou at the Okpara Breeding Farm in Benin is necessary for a better validation of the crossing schemes.

## Authors’ Contributions

FZAB and VA: Protocol writing, data collection, and manuscript writing. ITA and STS: Protocol validation and manuscript writing monitoring. AA, FZT, and AMY: Animals breeding, artificial insemination, and zootechnical and animal health monitoring. GAB and IOD: Data recording and processing. IYAK: Work coordination and article correspondent. All authors read and approved the final manuscript.
